# Farnesoid X receptor inhibits LNcaP cell proliferation via the upregulation of PTEN

**DOI:** 10.3892/etm.2014.1894

**Published:** 2014-08-11

**Authors:** JUN LIU, SHI-JUN TONG, XIANG WANG, LIAN-XI QU

**Affiliations:** Department of Urology Surgery, Huashan Hospital Affiliated to Fudan University, Shanghai 200040, P.R. China

**Keywords:** prostate cancer, farnesoid X receptor, PTEN, GW4064

## Abstract

Prostate cancer is a form of cancer that develops in the prostate, a gland in the male reproductive system. In the present study, the activation of the farnesoid X receptor (FXR), a member of the nuclear receptor superfamily, was demonstrated to inhibit cell proliferation in LNcaP cells. Using clinical samples, mRNA and protein levels of FXR were found to be significantly decreased by quantitative PCR and western blot analysis in prostate cancer tissues. *In vitro* studies identified further that activation or overexpression of FXR suppressed prostate cancer cell proliferation as measured by BrdU incorporation assays. At the molecular level, the results further revealed that the expression of the tumor suppressor gene, PTEN, was upregulated by FXR activation. Therefore, the observations indicated that FXR functions as a tumor suppressor in prostate cancer, which may provide a novel method for molecular targeting cancer treatment.

## Introduction

The farnesoid X receptor (FXR), a member of the nuclear receptor superfamily, was initially isolated in the liver and identified as a bile acid sensor (1–5). FXR plays a critical role in the regulation of bile acid, cholesterol, triglyceride and glucose homeostasis (6–9). For example, ablation of the FXR in C57BL/6 mice was shown to result in severe hepatic cholestasis, liver steatosis and insulin resistance (6,9).

Previous studies have also hypothesized that FXR is involved in the regulation of tumorigenesis (10–13). One study demonstrated that male and female FXR knockout mice spontaneously developed liver tumors, which was accompanied with liver injury and inflammation (10). Loss of the FXR in ApcMin/^+^ and chronic colitis mouse models of intestinal tumorigenesis was shown to result in early mortality and increased tumor progression via the promotion of Wnt signaling by infiltrating neutrophils and macrophages and proinflammatory cytokine production (11). In addition, FXR agonists have been shown to reduce liver and intestine tumor growth and metastasis in an orthotopic mouse xenograft model (12). Furthermore, downregulation of FXR has been associated with multiple malignant clinicopathological characteristics in human hepatocellular carcinoma (13), indicating that FXR functions as an important tumor suppressor. However, whether FXR affects prostate cancer cell proliferation remains unknown. The aim of the present study was to investigate the roles and molecular mechanisms of FXR in prostate cancer cell proliferation.

## Materials and methods

### Cell culture and tissue samples

LNcaP cells were purchased from the American Type Culture Collection (Rockville, MD, USA). Cells were culture in Dulbecco’s modified Eagle’s medium (DMEM; Gibco-BRL, Beijing, China) supplemented with 10% fetal bovine serum (Gibco-BRL). After seeding in the 96- or 6-well plates for 24 h, cells were treated with chenodeoxycholic acid (CDCA) (5 μM), GW4064 (2 μM) or vehicle control (DMSO). Small interfering RNA oligos targeting FXR or negative control (NC) were obtained from Genepharm Company (Shanghai, China). For the cell transfection experiments, LNcaP cells were grown to 70–80% confluence in six-well plates. The cells were transiently transfected using Lipofectamine 2000 (Invitrogen Life Technologies, Carlsbad, CA, USA). Prostate cancer tissues and adjacent normal tissues were collected from patients undergoing routine therapeutic surgery at the Department of Urology Surgery in Huashan Hospital Affiliated to Fudan University (Shanghai, China). All the samples were collected from patients that provided informed consent, and the experimental procedures were approved by the Institutional Review Board of Huashan Hospital Affiliated to Fudan University.

### mRNA isolation and quantitative polymerase chain reaction (PCR)

Total RNA was obtained from the tissue samples, and cells were harvested using TRIzol kits (Invitrogen Life Technologies). Quantitative PCR was performed using an Applied Biosystems 7900 Real-time PCR System (Shanghai, China) and a *Taq*Man Universal PCR Master Mix (Takara, Dalian, China), according to the manufacturer’s instructions.

### Bromodeoxyuridine (BrdU) assays

A cell proliferation enzyme-linked immunosorbent assay (ELISA; BrdU kit; Beyotime Institute of Biotechnology, Shanghai, China) was used to analyze the incorporation of BrdU during DNA synthesis, according to the manufacturer’s instructions. All the experiments were performed in triplicate, and the absorbance was measured at 450 nm using the Spectra Max 190 ELISA reader (Molecular Devices, Sunnyvale, CA, USA).

### Western blot analysis

Proteins were separated by 10% SDS-PAGE and transferred to nitrocellulose membranes (Amersham Bioscience, Little Chalfont, UK). Following blocking with 10% nonfat milk in phosphate-buffered saline, the membranes were immunoblotted with antibodies as indicated, followed by horseradish peroxidase-conjugated secondary antibodies (Cell Signaling Technology, Inc., Beverly, MA, USA). The signals were detected using a SuperSignal West Pico Chemiluminescent Substrate kit (Pierce Biotechnology, Inc., Rockford, IL, USA), according to manufacturer’s instructions. Anti-FXR, -PTEN and -Akt antibodies were purchased from Abcam (Cambridge, MA, USA). Protein expression levels of GAPDH were used as an internal control.

### Statistical analysis

Data are expressed as the mean ± standard error of the mean from at least three separate experiments. Differences between the groups were analyzed using the Student’s t-test, where P<0.05 was considered to indicate a statistically significant difference. Differences between the groups were analyzed by two-tailed Student’s t tests using SPSS version 13.0 (SPSS, Inc., Chicago, IL,USA).

## Results

### FXR activation inhibits cell proliferation

**Figure 1:**
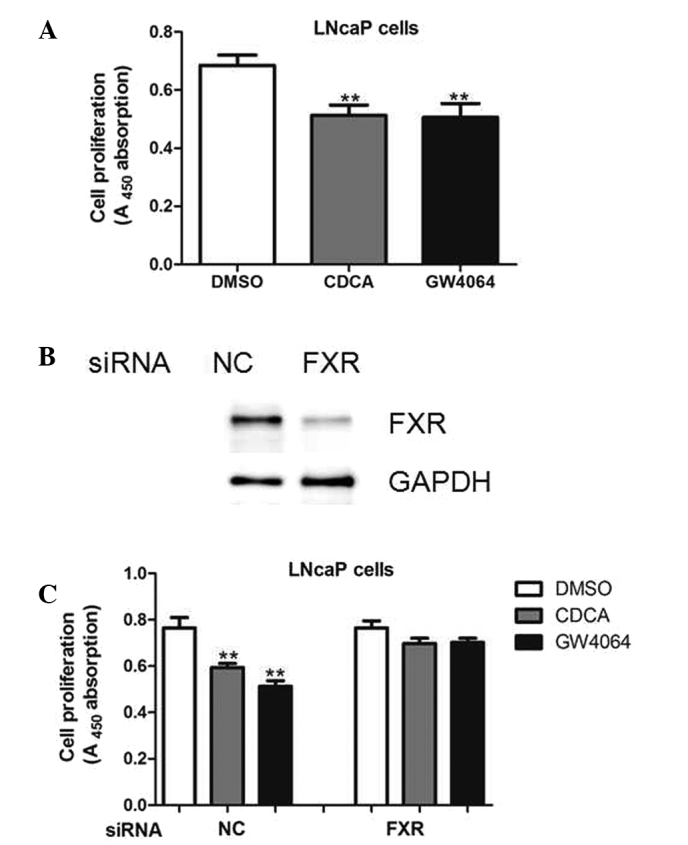
FXR activation inhibits prostate cancer cell proliferation. (A) LNcaP cells were treated with CDCA (5 μM), GW4064 (2 μM) or vehicle control (DMSO) and cell proliferation ability was measured using BrdU assays. A_450_ absorption was assayed following treatment for 24 h. (B) Western blot analysis showing FXR protein expression in the LNcaP cells transfected with siRNA oligos targeting FXR or a negative control. (C) LNcaP cells were pre-transfected with siRNA oligos targeting FXR or negative controls for 24 hr, and then treated with CDCA, GW4064 or vehicle control (DMSO) for BrdU cell proliferation assays. FXR, farnesoid X receptor; DMSO, dimethyl sulfoxide; CDCA, chenodeoxycholic acid; BrdU, bromodeoxyuridine; NC, negative control.

To evaluate the effects of FXR on prostate cancer cell growth, LNcaP cells were treated with the FXR agonists, CDCA and GW4064. As shown in Fig. 1, CDCA and GW4064 decreased the proliferative ability of LNcaP cells (Fig. 1). Next, endogenous FXR expression was silenced using specific small interfering RNA oligos in the LNcaP cells (Fig. 1B). As expected, CDCA and GW4064 were unable to exert antiproliferative roles in the presence of siRNA oligos targeting FXR (Fig. 1C), indicating that the antiproliferative roles of the two compounds were dependent on FXR expression.

### FXR overexpression represses LNcaP cell proliferation

**Figure 2:**
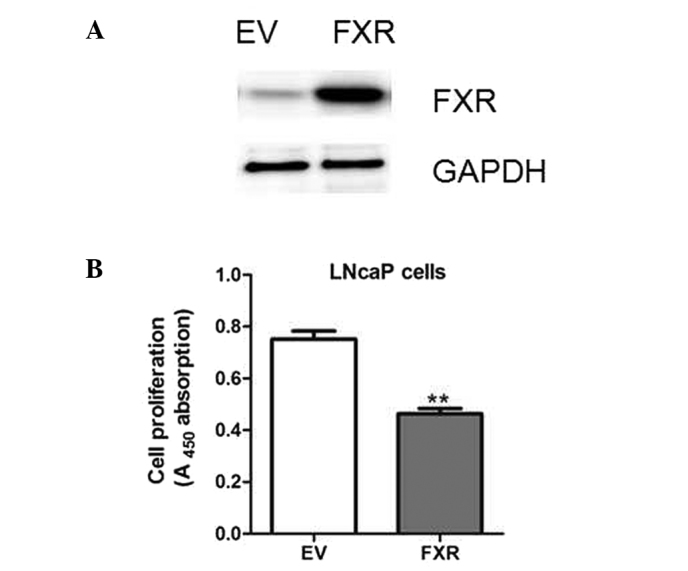
FXR overexpression inhibits prostate cancer cell proliferation. (A) FXR expression levels were determined by western blot analysis in LNcaP cells transfected with plasmids expressing an empty vector or FXR. (B) BrdU assays were used to determine the cell proliferative potential of LNcaP cells transfected with an empty vector or FXR. A_450_ absorption was assayed following transfection for 24 h. FXR, farnesoid X receptor; BrdU, bromodeoxyuridine; EV, empty vector.

To further determine the potential functions of the FXR, LNcaP cells were transfected with plasmids encoding FXR cDNA or an empty vector (Fig. 2A). As a result, FXR overexpression resulted in decreased cell proliferation, as measured by BrdU analysis (Fig. 2B). Therefore, the results indicated that FXR may be a tumor suppressor in prostate cancer cells.

### FXR upregulates the expression levels of the PTEN tumor suppressor

**Figure 3:**
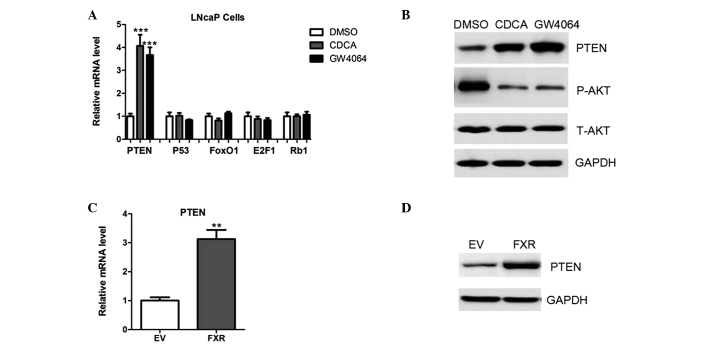
FXR upregulates the expression of PTEN. (A) mRNA expression levels of PTEN, p53, FOXO1, E2F1 and RB1 in LNcaP cells treated with a vehicle control (DMSO), CDCA or GW4064. (B) Protein expression levels of PTEN, phosphorylated and total Akt in LNcaP cells treated with a vehicle control (DMSO), CDCA or GW4064. (C) mRNA and (D) protein expression levels of PTEN in LNcaP cells transfected with plasmids expressing an empty vector or FXR. FXR, farnesoid X receptor; DMSO, dimethyl sulfoxide; CDCA, chenodeoxycholic acid; EV, empty vector; P-AKT, phosphorylated Akt; T-AKT, total Akt.

As FXR was shown to inhibit cell proliferation, the effects of the receptor on the expression of the genes associated with cell proliferation were investigated. Results from quantitative PCR analysis indicated that PTEN was highly upregulated following CDCA or GW4064 treatment, while other genes, including p53, FOXO1, E2F1 and RB1, remained unchanged (Fig. 3A). In addition, the upregulation of PTEN was confirmed by western blot analysis (Fig. 3B). Consistently, a reduction in the level of Akt phosphorylation was observed in the LNcaP cells treated with CDCA or GW4064 (Fig. 3B). Furthermore, PTEN expression was upregulated in the LNcaP cells transfected with FXR when compared with the cells transfected with the empty vectors (Fig. 3C and D).

### FXR expression levels are decreased in prostate cancer tissues

**Figure 4:**
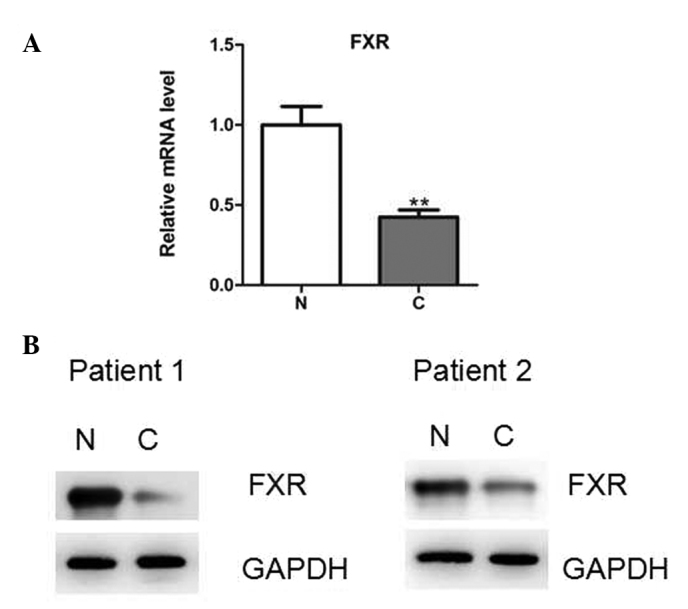
FXR expression levels in prostate cancer tissues. FXR expression levels were determined by (A) quantitative PCR and (B) representative western blot analysis in 25 pairs of human prostate cancer tissues and adjacent noncancerous tissues. FXR, farnesoid X receptor; PCR, polymerase chain reaction; C, cancer tissues; N, noncancerous tissues.

Finally, whether FXR was differentially expressed in human prostate cancer tissues was investigated. The mRNA and protein expression levels were determined using quantitative PCR and western blot analysis, respectively, in human prostate cancer tissues and pair-matched adjacent normal tissues. The results demonstrated that FXR expression was significantly decreased in the prostate cancer tissues (Fig. 4A and B).

## Discussion

In the present study, FXR activation or overexpression was demonstrated to inhibit cell proliferation in LNcaP cells. In addition, FXR expression was downregulated in prostate cancer tissues. Therefore, to the best of our knowledge, the present study, for the first time, identified that FXR may be a tumor suppressor in the progression of prostate cancer. However, the mechanisms underlying FXR downregulation remain unknown. Previous studies have demonstrated that glucose, insulin, proinflammatory cytokines and certain microRNAs are able to regulate FXR in a variety of tissues or cells (14–16). Therefore, further research into whether these factors contribute to the downregulation of FXR expression in prostate cancer should be performed.

Previous studies have demonstrated that FXR can protect against tumorigenesis and inhibit cell proliferation in several cancer types, including hepatocellular carcinoma and colon cancer (10–12). Through the induction of downstream target genes, such as SHP, FXR suppresses cell proliferation and promotes apoptosis (17). Accordingly, SHP null mice were shown to develop spontaneous liver tumors, and the expression of SHP was demonstrated to be downregulated in human cancer tissues (18,19).

In the present study, the results revealed that the expression of the tumor suppressor gene, PTEN, was upregulated following FXR activation. In humans, the loss or mutation of PTEN has been observed in a group of autosomal dominant syndromes, which are characterized by neurological disorders, multiple hamartomas and cancer susceptibility (20). In prostate cancer tissues, aberrant methylation of the PTEN gene has been observed, which resulted in the inactivation of PTEN and the hyperactivation of Akt (21). Therefore, the FXR/PTEN signaling pathway may be a novel pharmaceutical target for the treatment of prostate cancer.

In conclusion, the key observation of the present study is that FXR inhibits the proliferation of prostate cancer cell lines via the upregulation of PTEN expression. Understanding the precise role played by FXR is likely to advance the knowledge of prostate cancer biology, which may be beneficial for future treatment.
